# A basidomycetous hydroxynaphthalene-prenylating enzyme exhibits promiscuity toward prenyl donors

**DOI:** 10.1007/s00253-023-12621-1

**Published:** 2023-06-16

**Authors:** Andreas Martin, Nele Dierlamm, Georg Zocher, Shu-Ming Li

**Affiliations:** 1 Institut für Pharmazeutische Biologie und Biotechnologie, Fachbereich Pharmazie, Philipps-Universität Marburg, Robert-Koch Straße 4, 35037 Marburg, Germany; 2 Interfaculty Institute of Biochemistry (IFIB), University of Tübingen, Auf Der Morgenstelle 34, 72076 Tübingen, Germany

**Keywords:** Enzymatic synthesis, Prenyltransferase, ShPT, Hydroxynaphthalenes

## Abstract

**Abstract:**

The fungal prenyltransferase ShPT from *Stereum hirsutum* was believed to prenylate 4-hydroxybenzyl alcohol and thereby be involved in the vibralactone biosynthesis. In this study, we demonstrate that hydroxynaphthalenes instead of benzyl alcohol or aldehyde were accepted by ShPT for regular C-prenylation in the presence of both dimethylallyl and geranyl diphosphate. Although the natural substrate of ShPT remains unknown, our results provide one additional prenyltransferase from basidiomycetes, which are less studied, in comparison to those from other sources. Furthermore, this study expands the chemical toolbox for regioselective production of prenylated naphthalene derivatives.

**Key points:**

•*Basidiomycetous prenyltransferase*

•*Biochemical characterization*

•*A DMATS prenyltransferase prenylating hydroxynaphthalene derivatives*

**Supplementary Information:**

The online version contains supplementary material available at 10.1007/s00253-023-12621-1.

## Introduction

Enzymes involved in the biosynthesis of natural products have become a valuable resource for the discovery and generation of novel drug leads (Yi et al. 2021). One of these enzyme classes are prenyltransferases (PTs), which have been put in the spotlight of biotechnology and medicinal chemistry alike. PTs are found in every phylum of life and can catalyze an alkylation between an isoprenoid donor and an acceptor molecule. Thereby, they facilitate the formation of a new carbon–carbon, carbon–oxygen, or carbon–nitrogen bond. The isoprenoid moiety can be originated from dimethylallyl (DMAPP), geranyl (GPP), or farnesyl diphosphate (FPP). Acceptor molecules can be simple aliphatic and aromatic molecules, but can also include peptides, proteins, and t-RNAs in the primary or secondary metabolism (Liang et al. 2002; Li 2009; Heide 2009; Winkelblech et al. 2015). Molecules decorated with such moieties differ clearly in their biological activities from their non-prenylated congeners (Wollinsky et al. 2012). Prenylated hydroxynaphthalene derivatives, for example, have been shown to exhibit intriguing pharmacological and biological effects such as antibiotic or anti-inflammatory activities (Shin-Ya et al. 1990; Pathirana et al. 1992).

A large group of PTs, which accept aromatic substrates, are characterized by a specific protein fold of five repetitive ααββ (ABBA) elements named PT-barrels. In this fold the ten antiparallel β-strands generate a roomy lumen, in which the active site of the enzyme is located (Kuzuyama et al. 2005). This structure is also conserved between bacteria and fungi (Metzger et al. 2009). ABBA prenyltransferases can be divided into two main groups. The members of the CloQ/NphB group are found in fungi as well as in bacteria and prenylate-only aromatic substrates (Heide 2009; Haug-Schifferdecker et al. 2010). With an exception for NphB, they do not contain an aspartate-rich NDxxD motif and do not need divalent metal ions for their catalysis (Bonitz et al. 2011). CloQ from *Streptomyces roseochromogenes* attaches a prenyl moiety to 4-phenylpyruvic acid during the clorobiocin biosynthesis (Pojer et al. 2003), while NphB is involved in the naphtherpin biosynthesis (Kuzuyama et al. 2005). Members of the dimethylallyl tryptophan synthases (DMATS) superfamily belong to another main group. They are mostly found in ascomycetous fungi and only seldom in bacteria, like LtxC (Edwards and Gerwick 2004) or CymD (Schultz et al. 2010). DMATS PTs are independent of metal ions, but their activity can be strongly enhanced by the presence of Ca^2+^, Mg^2+^, or other divalent cations. They also do not have aspartate-rich motifs (Pockrandt et al. 2012). Many DMATS PTs like AnaPT (Yin et al 2009), FtmPT1 (Grundmann and Li 2005), and CdpC3PT (Yin et al. 2010) use tryptophan-containing cyclodipeptides as natural or best substrates. They show usually a broad substrate flexibility and can also catalyze prenylation of hydroxynaphthalenes (Yu et al. 2011), especially after enzyme engineering (Zhao et al. 2017). Since the first description of a DMATS in *Claviceps purpurea* (later identified to be *Claviceps fusiformis*) involved in ergot alkaloids biosynthesis (Tsai et al. 1995), more than 40 enzymes have been described and biochemically investigated in 20 years (Winkelblech et al. 2015). Identification of PTs from this enzyme family remains an interesting topic of investigation with increasing number of new members described (Nies and Li 2021; Xu et al. 2022; Li et al. 2023).

In comparison to the large number of PTs from ascomycetes, only two PTs from the DMATS superfamily have been described for fungi from the basidiomycetous order russulales so far. One of them, BypB from *Stereum* sp. strain BY1, is involved in the cloquetin biosynthesis and uses DMAPP as prenyl donor for orsellinic acid prenylation (Braesel et al. 2017). The second one, VibPT from *Boreostereum vibrans*, has been reported to be involved in the biosynthesis of the pancreatic lipase inhibitor vibralactone (Zhao et al. 2013, 2017; Yang et al. 2016; Feng et al. 2020). VibPT uses 4-hydroxybenzyl alcohol and 4-hydroxybenzaldehyde as substrates and catalyzes C-prenylation in the presence of DMAPP (Bai et al. 2020). VibPT shares a sequence identity of 91% with the putative aromatic prenyltransferase XP_007308836.1 from the basidiomycetous fungus *Stereum hirsutum* FP-91666 (Zhao et al. 2017, 2013), termed ShPT hereafter. ShPT shows a sequence identify of 67% to BypB (Braesel et al. 2017). ShPT is the only known annotated DMATS prenyltransferase in the genome of *Stereum hirsutum* FP-91666 (Floudas et al. 2012).

*Stereum hirsutum* has been shown to be important in the removal of phenolic waste due to its delignification possibilities (Benavides et al. 2022), and also to be a producer of diverse prenylated phenols (Yun et al. 2002; Duan et al. 2015).

To understand the catalytic ability, we investigated ShPT biochemically. To our surprise, ShPT does not accept 4-hydroxybenzene alcohol or 4-hydroxybenzaldehyde as substrate. It uses naphthalene derivatives for prenylation. Product formation was detected for 10 hydroxynaphthalenes in the presence of both DMAPP and GPP with comparable efficiencies. Isolation and structural elucidation with NMR and MS analyses led to identification of nine prenylated products with three not described before.

## Materials and methods

### Chemicals

DMAPP and GPP were prepared according to the method described previously (Woodside et al. 1988). Naphthalene derivatives of the highest available purity were bought from Acros Organics, Alfa Aesar, Fluka, Sigma-Aldrich, Roth, and VWL.

### Sequence and phylogenetic analysis

Phylogenetic and sequence analysis were done according to the method described previously (Peter et al. 2022).

### Protein overproduction and purification

The coding sequence of ShPT without introns was synthesized with codon optimization for expression in *Escherichia coli* (available in GenBank under the accession number OQ925395) and cloned into the pET28a ( +) vector by BioCat GmbH (Heidelberg, Germany). Protein overproduction and purification were carried out in *E. coli* BL21 (DE3), as described previously (Zheng et al. 2020; Steffan et al. 2007). IPTG was added to a final concentration of 0.8 mM and the cells were then grown at 18 °C for 16 h.

### Enzyme activity tests

Enzyme activity was tested in 50 μl reaction mixtures containing 50 mM Tris–HCl, 10 mM CaCl_2_, 5% (v/v) DMSO, 1 mM naphthalene derivative, 1 mM DMAPP or GPP, and 1 μg purified protein. The reactions were carried out at 37 °C for 2 h and terminated by addition of 50 μl ice cold methanol. The assay mixtures were centrifuged at 13,300 rpm for 5 min and the supernatants were analyzed on HPLC. The product yields in triplicate assays were calculated by UV detection at 290 nm with isolated compounds as standards.

### Determination of kinetic parameters

The linearity of the ShPT reactions toward naphthalene derivatives was determined up to 30 min with 1 µg protein. For the determination of kinetic parameters of ShPT, triplicates of 50 μl assays contained 50 mM Tris–HCl, 10 mM CaCl_2_, 5% (v/v) DMSO, 1 mM DMAPP or GPP, 1 μg purified protein, and the naphthalene derivative. The initial concentrations of the substrate were 0.01 mM, 0.02 mM, 0.05 mM, 0.1 mM, 0.2 mM, 0.5 mM, 1 mM, 2 mM, and 5 mM. Assays were incubated for 15 min at 37 °C and stopped with 50 μl ice cold methanol. The samples were centrifuged at 13,300 rpm for 5 min and the supernatant was analyzed via HPLC. The assays with DMAPP and GPP were tested under the same conditions. Kinetic parameters were determined by using GraphPad Prism software with nonlinear regression tools.

### Isolation of the enzyme products and HPLC conditions

For product isolation, enzyme assays were scaled up to 20 ml, containing 50 mM Tris–HCl, 10 mM CaCl_2_, 5% (v/v) DMSO, 1 mM DMAPP or GPP, 1 mM naphthalin derivative, and 0.5 mg of purified enzyme at the beginning. The reaction mixtures were incubated for 6 h, while every 2 h, 0.25 mg of purified enzyme was added. After extraction thrice with 18 ml ethyl acetate, the organic phases were combined and concentrated in a rotary evaporator at 36 °C. The dried residues were then dissolved in 0.5 ml methanol and injected into HPLC.

For isolation, an Agilent HPLC 1260 series with a semi-preparative Agilent ZORBAX Eclipse XDB C18 HPLC column (9.4 × 250 mm, 5 μm) was used. Water (solvent A) and acetonitrile (solvent B) were used as solvents with a linear gradient of 10–100% (*v*/*v*) B at a flow rate of 2 ml/min in 20 min. The column was then washed with 100% (*v*/*v*) B at a flow rate of 2 ml/min for 5 min and equilibrated with 10% (*v*/*v*) B at a flow rate of 2 ml/min for 5 min.

### LC–MS analysis

LC–MS analysis was performed as described previously (Zhou and Li 2021). A linear gradient from 5 to 100% B was used at a flow rate of 0.25 ml/min for 10 min.

### NMR spectroscopic analysis

NMR data was recorded on a JEOL ECA-500 MHz spectrometer (JEOL, Tokyo, Japan) (Zhou and Li 2021). Data was processed using MestreNova 14.2.1 (Mestrelab Research, Santiago de Compostella, Spain) and chemical shifts were referred to the corresponding solvent signals, 3.34 ppm for CD_3_OD and 7.26 ppm for CDCl_3_. NMR data are listed in Tables S1–S5 and spectra are given as Figs. S2–S10.

## Results

### Sequence analysis

The genome of *Stereum hirsutum* FP-91666 SS1 was sequenced in 2012 (Floudas et al. 2012). The genomic sequence of the putative aromatic prenyltransferase ShPT with a length of 2039 bp contains nine exons disrupted by eight introns, which was predicted by RefSeq (O’Leary et al. 2016). The lengths of the exon sequences are 34 bp, 173 bp, 81 bp, 203 bp, 194 bp, 287 bp, 281 bp, 129 bp, and 145 bp, while the intron sequences are 57 bp, 62 bp, 70 bp, 63 bp, 64 bp, 59 bp, 68 bp, and 69 bp long, respectively. The deduced polypeptide of ShPT comprises 509 amino acids with a calculated molecular weight of 56.88 kDa.

**Fig. 1:**
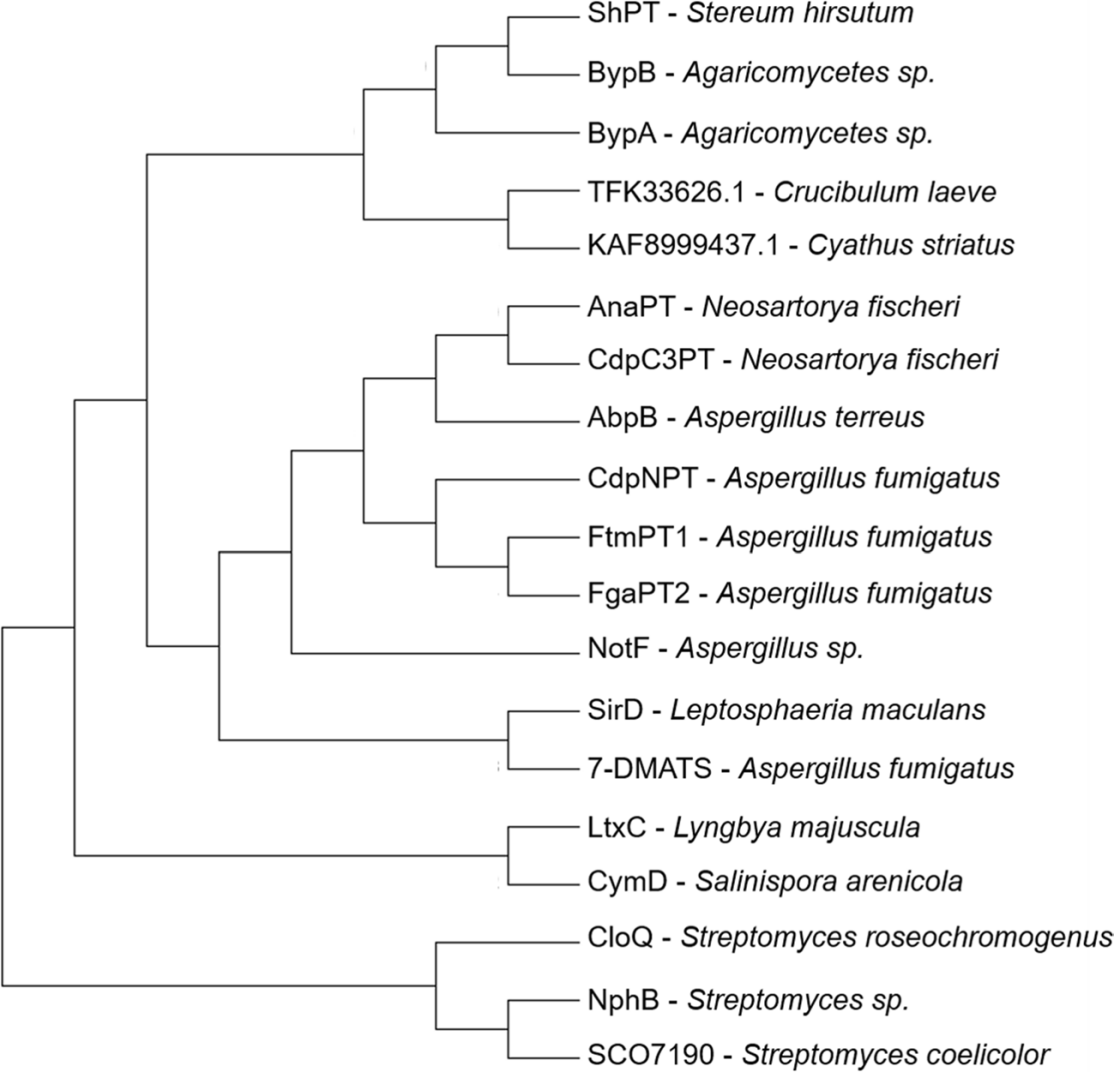
Phylogenetic analysis of ShPT and other PTs. Protein sequences were downloaded from NCBI database

A BLASTp search and multiple sequence alignment reveal that ShPT has very low homology to bacterial prenyltransferases of the CloQ/NphB type and shows only 21–36% sequence identities to DMATS prenyltransferases like CdpC3PT and AnaPT (Yin et al. 2009, 2010) from ascomycetes. Phylogenetic analysis reveals the location of basidomycetous, ascomycetous, and bacterial PTs in different clades (Fig. 1).

### Cloning, expression, and protein purification

**Fig. 2:**
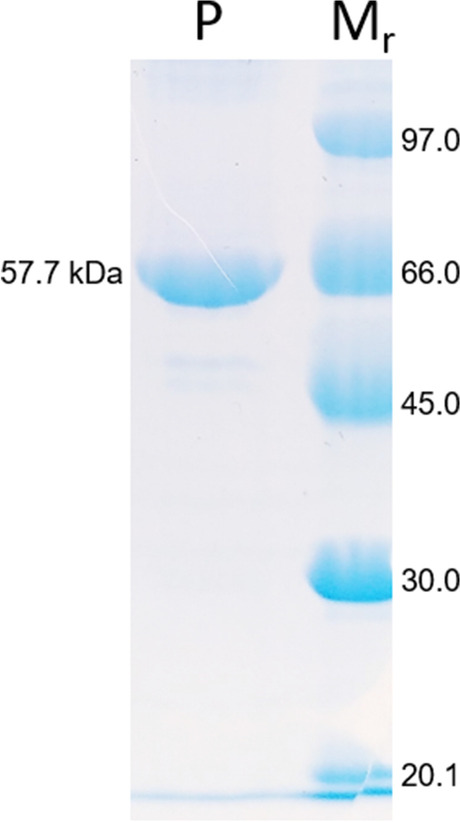
Analysis of overproduced and purified ShPT by SDS-PAGE (12%), stained with Coomassie brilliant blue R-250

pET28a ( +) vector with the codon-optimized coding sequence of ShPT was used for protein overproduction in *Escherichia coli* BL21 (DE3) cells. The recombinant His-tagged protein with a molecular weight of 57.7 kDa was purified on Ni–NTA agarose to near homogeneity (Fig. 2). A protein yield of 5.1 mg per liter culture was calculated.

### Substrate specificity of ShPT

**Fig. 3:**
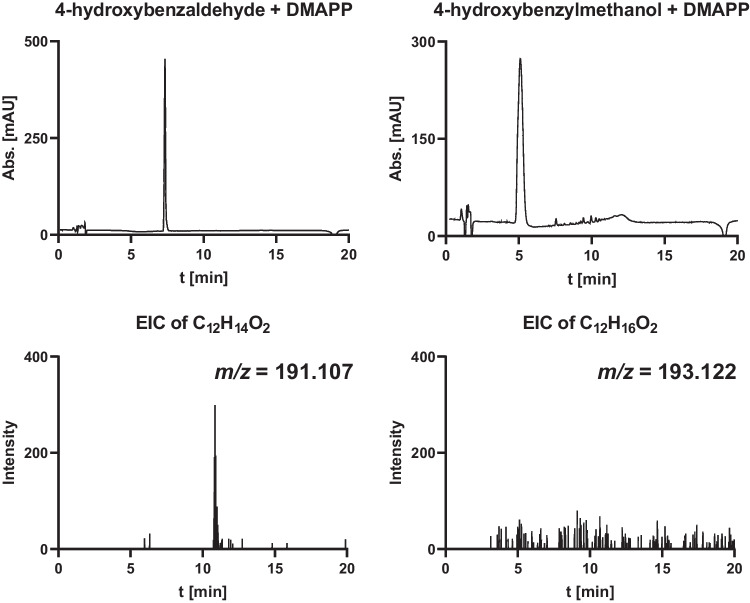
LC–MS chromatograms of reaction mixtures of ShPT with 4-hydroxybenzaldehyde and 4-hydroxybenzyl alcohol. UV absorptions at 280 nm are illustrated. Extracted ion chromatography (EIC) of [M + H]^+^ of the presumed prenylated product with a tolerance range of ± 0.005

For initial activity testing, we used 4-hydroxybenzyl alcohol and 4-hydroxybenzaldehyde as substrates and chose assay conditions described for VibPT (Bai et al. 2020). As shown in Fig. 3, no product could be detected by LC–MS under UV in the assays of both 4-hydroxybenzyl alcohol and 4-hydroxybenzaldehyde. Only trace amount of presumed product was observed for 4-hydroxybenzaldehyde in the extracted ion chromatogram (Fig. 3). It seems that both compounds are not natural substrates of ShPT.

**Fig. 4:**
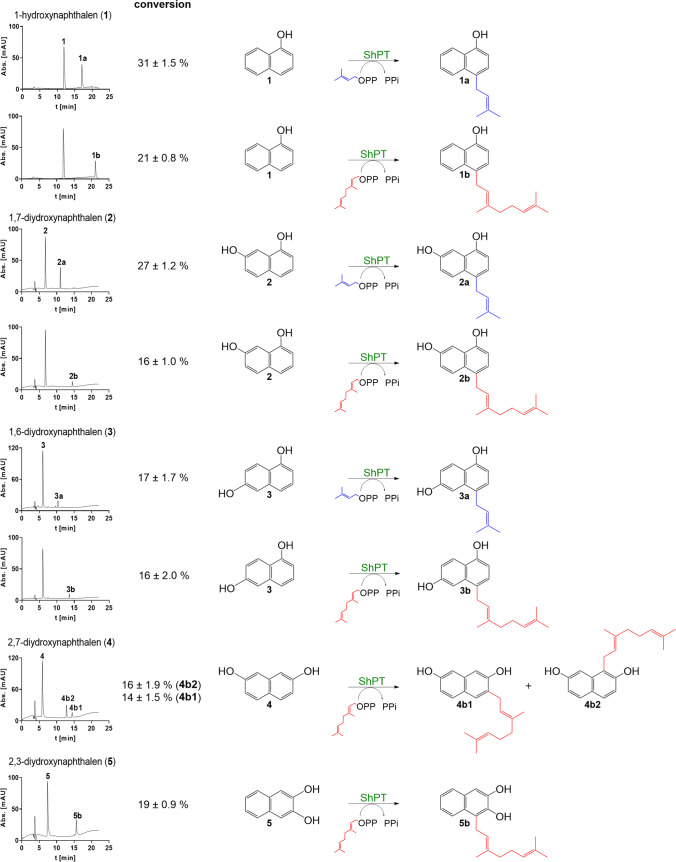
HPLC analysis of reaction mixtures of **1**–**5** with DMAPP and GPP as well as the prenyl transfer reactions. Absorptions were monitored at 295 nm. All assays were performed in triplicates and conversion yields are given as mean value with standard error

Based on the low-sequence identity to FtmPT1 and the prediction of an ABBA-type PT domain in silico, we tested the ShPT activity toward 1-hydroxynaphthalene (1-naphthol, **1**) in the presence of DMAPP and GPP. As shown in Fig. 4, 31 ± 1.5 and 21 ± 0.8% of **1** were converted to its prenylated products after incubation with 5 µg of ShPT and 1 mM DMAPP or GPP for 12 h at 37 °C. Additional tests indicated the acceptance of nine hydroxynaphthalenes in the presence of DMAPP and seven in the presence of GPP (Figs. 4 and S1). Product yields between 27 ± 1.2 and 0.2 ± 0.1% were calculated for the assays of DMAPP with 1,7- (**2**) and 1,6-dihydroxynaphthalene (**3**) as the best accepted substrates. In the presence of GPP, product yields between 30 ± 1.9 and 1.8 ± 0.9% were detected with 2,7-dihydroxynaphthalene (**4**) as the best substrate (Figs. 4 and S1). The product formation was strictly dependent on the presence of ShPT as well as DMAPP or GPP and could not be detected in the negative controls (data not shown).

### Structure elucidation and confirmation of the prenylation position

Detailed inspection of the HPLC chromatograms revealed that one predominant product was formed in most of the enzyme assays (Figs. 4 and S1). To elucidate the structure and understand the prenylation position, the enzyme products of **1** − **3** with DMAPP and GPP as well as those of (**4**) and 2,3-dihydroxynaphthalene (**5)** with GPP were isolated from 20 ml incubation mixtures on a semi-preparative scale HPLC. The obtained products were subjected to LCMS and NMR analyses. Positive HR-ESI–MS data proved the attachment of a dimethylallyl residue in the product **a** series from DMAPP assays and a geranyl moiety in the product **b** series from GPP assays (Table S1), which was deducted from their 68 and 136 Da higher masses than the corresponding substrate, respectively.

This conclusion is also confirmed by the NMR data. For products from the DMAPP reactions, signals belonging to a dimethylallyl moiety are observed at δ_H_ 1.5–1.8 (two s, 3H-4′ and 3H-5′), 3.5–3.6 (d, 2H-1′), and 5.3–5.4 ppm (brt, H-2′). The signals of the geranylated products appear at δ_H_ 1.5–1.9 (three s, 3H-4′, 3H-9′, and 3H-10′), 1.9–2.2 (m, 2H-5′, 2H-6′), 3.3–3.7 (d, 2H-1′), and 5.0–5.4 (two brt, H-2′, H-7′). In the ^1^H-NMR spectra of **1a**, **1b**, **2a**, **2b**, **3a**, **3b**, and **5b**, the signals for H-4 disappeared, showing signals for only six instead of seven protons in the aromatic region of **1a** and **1b** and five instead of six for **2b**, **3a**, **3b**, and **5b**. Interpretation of the coupling pattern proved the attachment of the prenyl residues in **1a**, **1b**, **2a**, **2b**, **3a**, and **3b** at the *para-*position of the 1-hydroxyl group (Tables S2–S4, Figs. S2–S7) and in **5b** at the *ortho*-position (Table S6, Fig. S10). Coupling pattern also provided evidence for the attachment of the geranyl group at an *ortho*-position in **4b1** and **4b2** (Table S5, Figs. S8 and S9).

### Kinetic parameters of ShPT

The kinetic parameters of three of the best substrates, i.e., **1**–**3**, were determined to get a better insight into the catalytic efficiency of the enzyme. Therefore, the Michaelis–Menten constant (*K*_M_) and turnover number (*k*_cat_) were calculated from Hanes-Woolf and Lineweaver-Burke plots (Table 1, Figs. S11–S15).

**Table 1:** Kinetic parameters of ShPT

Substrate	*K*_M_ [mM]	*k*_cat_ [s^−1^]	*k*_cat_/*K*_M_ [s^−1^ M^−1^]
1-hydroxynaphthalen (**1**)	0.72 ± 0.02	0.38 ± 0.02	521
1,7-dihydroxynaphthalen (**2**)	0.92 ± 0.01	0.46 ± 0.04	511
1,6-dihydroxynaphthalen (**3**)	0.66 ± 0.10	0.13 ± 0.01	197
DMAPP	0.55 ± 0.02	0.19 ± 0.02	345
GPP^a^	1.01 ± 0.05	0.36 ± 0.01	357

The mean values of three independent experiments are given with standard error

The kinetic parameters of **1**–**3** were determined in the presence of DMAPP. **1** was used as co-substrate for DMAPP and GPP reactions

^a^Substrate inhibition at 1.0 mM or higher concentrations

The *K*_M_ values of ShPT toward hydroxynaphthalenes in the range of 0.66 to 0.92 are comparable. The reactions with **1** and **2** have significant higher turnover numbers than **3**, so that more than twofold catalytic efficiencies were calculated (Table 1). ShPT shows a higher affinity, but lower turnover number toward DMAPP than GPP. As a consequence, comparable catalytic efficiencies were obtained for DMAPP and GPP in the presence of **1** (Table 1).

## Discussion

In summary, we proved in this study the putative prenyltransferase ShPT as the first hydroxynaphthalene-prenylating enzyme from basidiomycetes. To our surprise, although ShPT shares a sequence identity of 91% with VibPT on the amino acid level, these enzymes catalyze prenylation of different substance classes. ShPT shows low-sequence identity to cyclodipeptide prenyltransferases CdpC3PT and AnaPT from ascomycetes, which also accepted hydroxynaphthalenes as prenylation substrates (Yu et al. 2011). Biochemical investigation proved that both DMAPP and GPP are comparable good prenyl donors for ShPT. In the presence of DMAPP, ShPT with a catalytic efficiency of 521 s^−1^ M^−1^ for 1-naphthol is comparable to AnaPT (569 s^−1^ M^−1^) and more efficient than CdpC3PT (238 s^−1^ M^−1^) or 7-DMATS (46 s^−1^ M^−1^) (Yu et al. 2011). The efficiency of ShPT with GPP and 1-naphthol (357 s^−1^ M^−1^) is much higher than those of NphB with different hydroxynaphthalenes (0.3–7.7 s^−1^ M^−1^ (Kumano et al. 2008)). It becomes apparent that ShPT is a good candidate as a hydroxynaphthalene-prenylating enzyme for further study. It would be interesting to solve the crystal structure of ShPT to get further insights into its catalyzing mechanism and to improve its efficiency even by enzyme engineering, especially for non-natural substrates (Zhao et al. 2017).

## Supplementary Information

Below is the link to the electronic supplementary material.

Supplementary file1 (PDF 1687 KB)

## Data Availability

All data generated during this study are included in this published article and its supplementary information file.

## References

[CR1] Bai N, Li GH, Luo SL, Du L, Hu QY, Xu HK, Zhang KQ, Zhao PJ (2020) Vib-PT, an aromatic prenyltransferase involved in the biosynthesis of vibralactone from *Stereum vibrans*. Appl Environ Microbiol 86:e02687-e2719. 10.1128/AEM.02687-19 10.1128/AEM.02687-19 PMC7205494 32144102

[CR2] Benavides V, Pinto-Ibieta F, Serrano A, Rubilar O, Ciudad G (2022) Use of *Anthracophyllum discolor* and *Stereum hirsutum* as a suitable strategy for delignification and phenolic removal of olive mill solid waste. Foods 11:1587. 10.3390/foods11111587 10.3390/foods11111587 PMC9180551 35681337

[CR3] Bonitz T, Alva V, Saleh O, Lupas AN, Heide L (2011) Evolutionary relationships of microbial aromatic prenyltransferases. PLoS One 6:e27336. 10.1371/journal.pone.0027336 10.1371/journal.pone.0027336 PMC3227686 22140437

[CR4] Braesel J, Fricke J, Schwenk D, Hoffmeister D (2017) Biochemical and genetic basis of orsellinic acid biosynthesis and prenylation in a stereaceous basidiomycete. Fungal Genet Biol 98:12–19. 10.1016/j.fgb.2016.11.007 10.1016/j.fgb.2016.11.007 27903443

[CR5] Duan Y-C, Meng X-X, Yang Y-L, Yang Y-H, Zhao P-J (2015) Two new phenol derivatives from *Stereum hirsutum* FP-91666. J Asian Nat Prod Res 17:324–328. 10.1080/10286020.2014.959439 10.1080/10286020.2014.959439 25295617

[CR6] Edwards DJ, Gerwick WH (2004). Lyngbyatoxin biosynthesis: sequence of biosynthetic gene cluster and identification of a novel aromatic prenyltransferase. J Am Chem Soc.

[CR7] Feng K-N, Yang Y-L, Xu Y-X, Zhang Y, Feng T, Huang S-X, Liu J-K, Zeng Y (2020). A hydrolase-catalyzed cyclization forms the fused bicyclic β-lactone in vibralactone. Angew Chem Int Ed Engl.

[CR8] Floudas D, Binder M, Riley R, Barry K, Blanchette RA, Henrissat B, Martínez AT, Otillar R, Spatafora JW, Yadav JS, Aerts A, Benoit I, Boyd A, Carlson A, Copeland A, Coutinho PM, de Vries RP, Ferreira P, Findley K, Foster B, Gaskell J, Glotzer D, Górecki P, Heitman J, Hesse C, Hori C, Igarashi K, Jurgens JA, Kallen N, Kersten P, Kohler A, Kües U, Kumar TKA, Kuo A, LaButti K, Larrondo LF, Lindquist E, Ling A, Lombard V, Lucas S, Lundell T, Martin R, McLaughlin DJ, Morgenstern I, Morin E, Murat C, Nagy LG, Nolan M, Ohm RA, Patyshakuliyeva A, Rokas A, Ruiz-Dueñas FJ, Sabat G, Salamov A, Samejima M, Schmutz J, Slot JC, St John F, Stenlid J, Sun H, Sun S, Syed K, Tsang A, Wiebenga A, Young D, Pisabarro A, Eastwood DC, Martin F, Cullen D, Grigoriev IV, Hibbett DS (2012). The paleozoic origin of enzymatic lignin decomposition reconstructed from 31 fungal genomes. Science.

[CR9] Grundmann A, Li S-M (2005). Overproduction, purification and characterization of FtmPT1, a brevianamide F prenyltransferase from *Aspergillus fumigatus*. Microbiology.

[CR10] Haug-Schifferdecker E, Arican D, Brueckner R, Heide L (2010). A new group of aromatic prenyltransferases in fungi, catalyzing a 2,7-dihydroxynaphthalene dimethylallyltransferase reaction. J Biol Chem.

[CR11] Heide L (2009). Prenyl transfer to aromatic substrates: genetics and enzymology. Curr Opin Chem Biol.

[CR12] Kumano T, Richard SB, Noel JP, Nishiyama M, Kuzuyama T (2008). Chemoenzymatic syntheses of prenylated aromatic small molecules using *Streptomyces* prenyltransferases with relaxed substrate specificities. Bioorg Med Chem.

[CR13] Kuzuyama T, Noel JP, Richard SB (2005). Structural basis for the promiscuous biosynthetic prenylation of aromatic natural products. Nature.

[CR14] Li S-M (2009). Applications of dimethylallyltryptophan synthases and other indole prenyltransferases for structural modification of natural products. App Microbol Biotechnol.

[CR15] Li W, Coby L, Zhou J, Li S-M (2023). Diprenylated cyclodipeptide production by changing the prenylation sequence of the nature’s synthetic machinery. Appl Microbiol Biotechnol.

[CR16] Liang PH, Ko TP, Wang AH (2002). Structure, mechanism and function of prenyltransferases. Eur J Biochem.

[CR17] Metzger U, Schall C, Zocher G, Unsöld I, Stec E, Li S-M, Heide L, Stehle T (2009). The structure of dimethylallyl tryptophan synthase reveals a common architecture of aromatic prenyltransferases in fungi and bacteria. Proc Natl Acad Sci U S A.

[CR18] Nies J, Li S-M (2021). Prenylation and dehydrogenation of a *C2*-reversely prenylated diketopiperazine as a branching point in the biosynthesis of echinulin family alkaloids in *Aspergillus ruber*. ACS Chem Biol.

[CR19] O’Leary NA, Wright MW, Brister JR, Ciufo S, Haddad D, McVeigh R, Rajput B, Robbertse B, Smith-White B, Ako-Adjei D, Astashyn A, Badretdin A, Bao Y, Blinkova O, Brover V, Chetvernin V, Choi J, Cox E, Ermolaeva O, Farrell CM, Goldfarb T, Gupta T, Haft D, Hatcher E, Hlavina W, Joardar VS, Kodali VK, Li W, Maglott D, Masterson P, McGarvey KM, Murphy MR, O'Neill K, Pujar S, Rangwala SH, Rausch D, Riddick LD, Schoch C, Shkeda A, Storz SS, Sun H, Thibaud-Nissen F, Tolstoy I, Tully RE, Vatsan AR, Wallin C, Webb D, Wu W, Landrum MJ, Kimchi A, Tatusova T, DiCuccio M, Kitts P, Murphy TD, Pruitt KD (2016). Reference sequence (RefSeq) database at NCBI: current status, taxonomic expansion, and functional annotation. Nucleic Acids Res.

[CR20] Pathirana C, Jensen PR, Fenical W (1992). Marinone and debromomarinone: antibiotic sesquiterpenoid naphthoquinones of a new structure class from a marine bacterium. Tetrahedron Lett.

[CR21] Peter M, Yang Y, Li S-M (2022). A terpene cyclase from Aspergillus ustus is involved in the biosynthesis of geosmin precursor germacradienol. RSC Adv.

[CR22] Pockrandt D, Ludwig L, Fan A, König GM, Li S-M (2012). New insights into the biosynthesis of prenylated xanthones: XptB from *Aspergillus nidulans* catalyses an *O*-prenylation of xanthones. ChemBioChem.

[CR23] Pojer F, Wemakor E, Kammerer B, Chen H, Walsh CT, Li S-M, Heide L (2003) CloQ, a prenyltransferase involved in clorobiocin biosynthesis. Proc Natl Acad Sci USA 100:2316–2321. 10.1073/pnas.0337708100 10.1073/pnas.0337708100 PMC151338 12618544

[CR24] Schultz AW, Lewis CA, Luzung MR, Baran PS, Moore BS (2010). Functional characterization of the cyclomarin/cyclomarazine prenyltransferase CymD directs the biosynthesis of unnatural cyclic peptides. J Nat Prod.

[CR25] Shin-Ya K, Imai S, Furihata K, Hayakawa Y, Kato Y, Vanduyne GD, Clardy J, Seto H (1990). Isolation and structural elucidation of an antioxidative agent, naphterpin. J Antibiot (Tokyo).

[CR26] Steffan N, Unsöld IA, Li S-M (2007). Chemoenzymatic synthesis of prenylated indole derivatives by using a 4-dimethylallyltryptophan synthase from *Aspergillus fumigatus*. ChemBioChem.

[CR27] Tsai HF, Wang H, Gebler JC, Poulter CD, Schardl CL (1995). The *Claviceps purpurea* gene encoding dimethylallyltryptophan synthase, the committed step for ergot alkaloid biosynthesis. Biochem Biophys Res Commun.

[CR28] Winkelblech J, Fan A, Li S-M (2015). Prenyltransferases as key enzymes in primary and secondary metabolism. Appl Microbiol Biotechnol.

[CR29] Wollinsky B, Ludwig L, Hamacher A, Yu X, Kassack MU, Li SM (2012). Prenylation at the indole ring leads to a significant increase of cytotoxicity of tryptophan-containing cyclic dipeptides. Bioorg Med Chem Lett.

[CR30] Woodside AB, Huang Z, Poulter CD (1988). Trisammonium geranyl diphosphate. Org Synth.

[CR31] Xu Y, Li D, Wang W, Xu K, Tan G, Li J, Li S-M, Yu X (2022). Dearomative gem-diprenylation of hydroxynaphthalenes by an engineered fungal prenyltransferase. RSC Adv.

[CR32] Yang Y-L, Zhou H, Du G, Feng K-N, Feng T, Fu X-L, Liu J-K, Zeng Y (2016). A monooxygenase from *Boreostereum vibrans* catalyzes oxidative decarboxylation in a divergent vibralactone biosynthesis pathway. Angew Chem Int Ed Engl.

[CR33] Yi D, Bayer T, Badenhorst CPS, Wu S, Doerr M, Hohne M, Bornscheuer UT (2021). Recent trends in biocatalysis. Chem Soc Rev.

[CR34] Yin W-B, Grundmann A, Cheng J, Li S-M (2009). Acetylaszonalenin biosynthesis in *Neosartorya fischeri*: Identification of the biosynthetic gene cluster by genomic mining and functional proof of the genes by biochemical investigation. J Biol Chem.

[CR35] Yin W-B, Yu X, Xie X-L, Li S-M (2010). Preparation of pyrrolo[2,3-*b*]indoles carrying a β-configured reverse *C3*-dimethylallyl moiety by using a recombinant prenyltransferase CdpC3PT. Org Biomol Chem.

[CR36] Yu X, Xie X, Li S-M (2011). Substrate promiscuity of secondary metabolite enzymes: prenylation of hydroxynaphthalenes by fungal indole prenyltransferases. Appl Microbiol Biotechnol.

[CR37] Yun B-S, Cho Y, Lee I-K, Cho S-M, Lee TH, Yoo I-D (2002). Sterins A and B, new antioxidative compounds from *Stereum hirsutum*. J Antibiot (Tokyo).

[CR38] Zhao P-J, Yang Y-L, Du L, Liu J-K, Zeng Y (2013). Elucidating the biosynthetic pathway for vibralactone: a pancreatic lipase inhibitor with a fused bicyclic β-lactone. Angew Chem Int Ed Engl.

[CR39] Zhao W, Fan A, Tarcz S, Zhou K, Yin WB, Liu XQ, Li S-M (2017). Mutation on Gly115 and Tyr205 of the cyclic dipeptide *C2*-prenyltransferase FtmPT1 increases its catalytic activity toward hydroxynaphthalenes. Appl Microbiol Biotechnol.

[CR40] Zheng L, Wang H, Fan A, Li S-M (2020). Oxepinamide F biosynthesis involves enzymatic D-aminoacyl epimerization, 3H-oxepin formation, and hydroxylation induced double bond migration. Nat Commun.

[CR41] Zhou J, Li S-M (2021). Conversion of viridicatic acid to crustosic acid by cytochrome P450 enzyme-catalysed hydroxylation and spontaneous cyclisation. App Microbiol Biotechnol.

