# Supplementary material for: A basidomycetous hydroxynaphthalene-prenylating enzyme exhibits promiscuity toward prenyl donors
Source: Appl Microbiol Biotechnol. 2023 Jun 16;107(15):4845–52. doi: 10.1007/s00253-023-12621-1 (PMC10344970; doi:10.1007/s00253-023-12621-1)
Supplement: Supplementary file 1 — Supplementary file1 (PDF 1687 KB) [file 253_2023_12621_MOESM1_ESM.pdf]

Supporting Information

A basidomycetous hydroxynaphthalene-prenylating enzyme exhibits promiscuity towards prenyl donors

Andreas Martin[1], Nele Dierlamm[2], Georg Zocher[2] and Shu-Ming Li[1*]

[1]Institut für Pharmazeutische Biologie und Biotechnologie, Fachbereich Pharmazie, Philipps-Universität Marburg, Robert-Koch Straße 4, 35037 Marburg, Germany

[2]Interfaculty Institute of Biochemistry (IFIB), University of Tübingen, Auf der Morgenstelle 34, 72076 Tübingen, Germany

Corresponding Author

*Tel +49 6421 28 22461. shuming.li@staff.uni-marburg.de

ORCID Shu-Ming Li: 0000-0003-4583-2655

Table of contents

Supplementary Tables 3

Table S1 HR-ESI-MS data of the reported compounds 3

Table S2 ^{1}H-NMR data of compounds **1a** and **1b** 4

Table S3 ^{1}H-NMR data of compounds **2a** and **2b** 5

Table S4 ^{1}H-NMR data of compounds **3a** and **3b** 6

Table S5 ^{1}H-NMR data of compounds **4b1** and **4b2** 7

Table S6 ^{1}H-NMR data of compound **5b** 8

Supplementary Figures 9

Fig. S1 LC-MS analysis of enzyme assays 9

Fig. S2 ^{1}H NMR spectrum of **1a** in CD_3OD (500 MHz) 10

Fig. S3 ^{1}H NMR spectrum of **1b** in $CDCl_3$ (500 MHz) 10

Fig. S4 ^{1}H NMR spectrum of **2a** in CD_3OD (500 MHz) 11

Fig. S5 ^{1}H NMR spectrum of **2b** in CD_3OD (500 MHz) 11

Fig. S6 ^{1}H NMR spectrum of **3a** in CD_3OD (500 MHz) 12

Fig. S7 ^{1}H NMR spectrum of **3b** in CD_3OD (500 MHz) 12

Fig. S8 ^{1}H NMR spectrum of **4b1** in CD_3OD (500 MHz) 13

Fig. S9 ^{1}H NMR spectrum of **4b2** in CD_3OD (500 MHz) 13

Fig. S10 ^{1}H NMR spectrum of **5b** in CD_3OD (500 MHz) 14

Fig. S11 Kinetic parameters of 1-hydroxynaphthol (**1**) with DMAPP as donor 14

Fig. S12 Kinetic parameters of 1,7-dihydroxynaphthalen (**2**) with DMAPP as donor 15

Fig. S13 Kinetic parameters of 1,6-dihydroxynaphthalen (**3**) with DMAPP as donor 15

Fig. S14 Kinetic parameters of DMAPP with 1-hydroxynaphthol (**1**) as acceptor 16

Fig. S15 Kinetic parameters of DMAPP with 1-hydroxynaphthol (**1**) as acceptor 16

References 17

Table S1 HR-ESI-MS data of the reported compounds

Compound	Formula	$[M + H]^+$ Calculated	$[M + H]^+$ Measured	Deviation [ppm]
**1a**	$C_{15}H_{17}O$	213.1274	213.1267	3.3
**1b**	$C_{20}H_{25}O$	281.1905	281.1901	1.4
**2a**	$C_{15}H_{17}O_2$	229.1223	229.1219	1.7
**2b**	$C_{20}H_{25}O_2$	297.1849	297.1844	1.7
**3a**	$C_{15}H_{17}O_2$	229.1223	229.1230	-3.1
**3b**	$C_{20}H_{25}O_2$	297.1849	297.1836	4.4
**4b1**	$C_{20}H_{25}O_2$	297.1849	297.1836	4.4
**4b2**	$C_{20}H_{25}O_2$	297.1849	297.1844	1.7
**5b**	$C_{20}H_{25}O_2$	297.1849	297.1848	0.3

Table S2: ^{1}H-NMR data of compounds **1a** and **1b**

Comp.	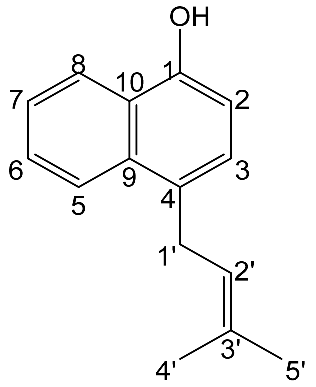 **1a**	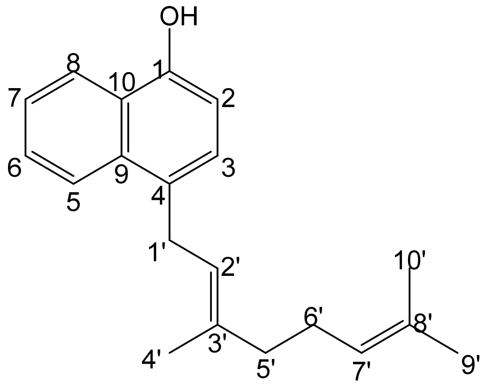 **1b**
Pos.	δ_H, multi, *J* in CD_3OD	δ_H, multi, *J* in $CDCl_3$
2	6.72, d, 7.6	6.75, d, 7.6
3	7.09, d, 7.6	7.15, d, 7.6
5	7.91, d, 8.4	7.97, d, 8.2
6	7.41, ddd, 8.4, 6.9, 1.4	7.47, t, 7.5
7	7.46, ddd, 8.4, 6.9, 1.4	7.52, t, 7.5
8	8.21, d, 8.4	8.22, d, 8.2
1'	3.65, d, 7.0	3.69, d, 6.9
2'	5.33, brt, 7.0	5.09, t, 6.8
4'	1.80, s	1.77, s
5'	1.74, s	2.06, m
6'	/	2.11, m
7'	/	5.38, t, 7.1
9'	/	1.66, s
10'	/	1.59, s

The NMR data of **1a** and **1b** correspond well to those published previously (Yu et al. 2011; Törincsi et al. 2012)

Table S3: ^{1}H-NMR data of compounds **2a** and **2b**

Comp.	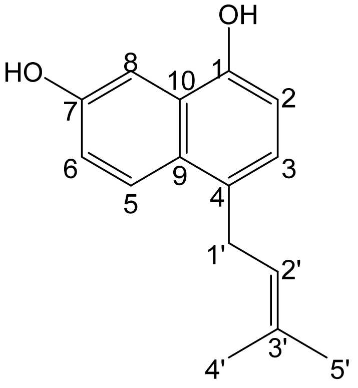<br>**2a**	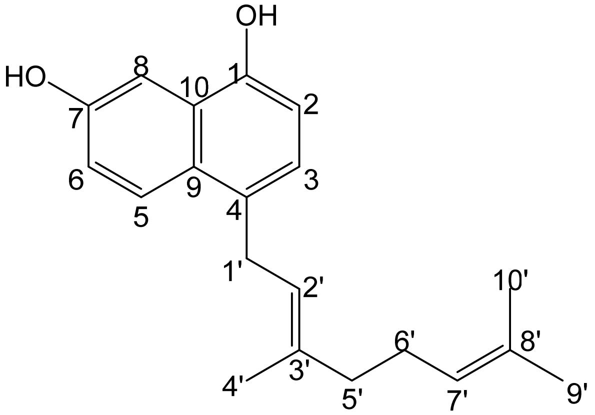<br>**2b**
Pos.	δ_H, multi, *J* in CD_3OD	δ_H, multi, *J* in CD_3OD
2	6.63, d 7.6	6.64, d 7.6
3	6.87, d, 7.6	6.89, d, 7.6
5	7.77, d, 8.9	7.77, d, 9.0
6	7.03, dd, 8.9, 2.6	7.04, dd, 9.0, 2.7
8	7.48, d, 2.6	7.49, d, 2.7
1'	3.59, d, 7.0	3.60, d, 7.0
2'	5.31, brt, 7.0	5.31, brt, 7.0
4'	1.78, s	1.77, s
5'	1.73, s	2.06, m
6'	/	2.11, m
7'	/	5.08, brt, 7.2
9'	/	1.63, s
10'	/	1.57, s

The NMR data of **2a** correspond well to those published previously (Yu et al. 2011)

Table S4: ^{1}H-NMR data of compounds **3a** and **3b**

Comp.	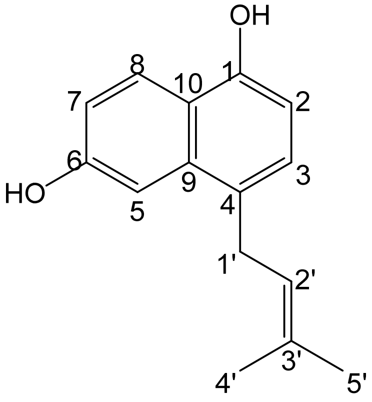 **3a**	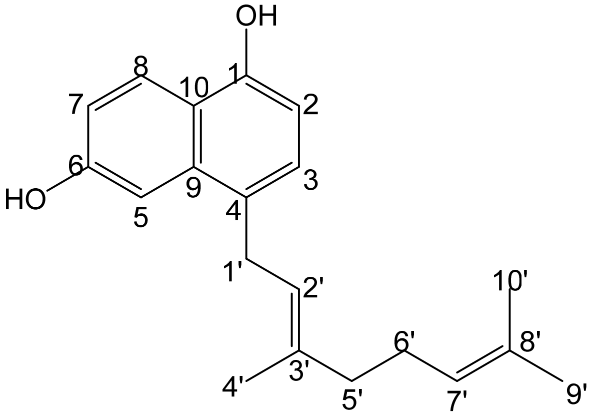 **3b**
Pos.	δ_H, multi, *J* in CD_3OD	δ_H, multi, *J* in CD_3OD
2	6.49, d, 7.6	6.51, d, 7.6
3	6.97, d, 7.6	7.01, d, 7.6
5	7.15, d, 2.5	7.18, d, 2.4
7	6.95, dd, 9.0, 2.5	6.98, dd, 9.0, 2.4
8	8.04, d, 9.0	8.07, d, 9.0
1'	3.51, d, 7.0	3.54, d, 7.0
2'	5.30, brt, 7.0	5.33, brt, 7.0
4'	1.77, s	1.77, s
5'	1.72, s	2.05, m
6'	/	2.12, m
7'	/	5.10, brt, 7.2
9'	/	1.62, s
10'	/	1.57, s

The NMR data of **3a** and **3b** correspond well to those published previously (Yu et al. 2011; Kumano et al. 2008)

Table S5: ^{1}H-NMR data of compounds **4b1** and **4b2**

Comp.	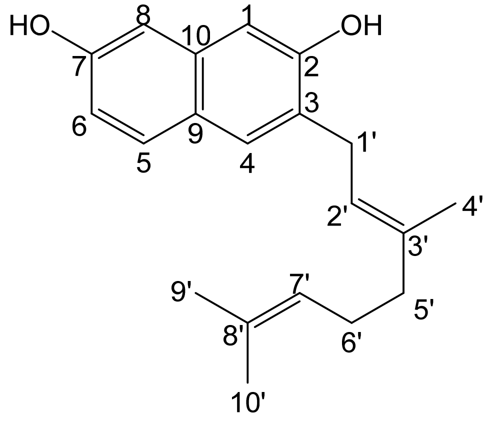 **4b1**	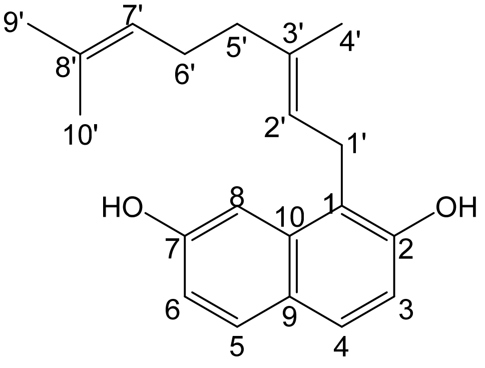 **4b2**
Pos.	δ$_H$, multi, *J* in CD_3OD	δ$_H$, multi, *J* in CD_3OD
1	7.34, s	/
3	/	6.87, d, 8.8
4	6.85, s	7.44, d, 8.8
5	7.46, d, 8.7	7.57, d, 8.6
6	6.77, dd, 8.7, 2.4	6.83, dd, 8.6, 2.0
8	6.83, d, 2.4	7.12, d, 2.0
1'	3.37, d, 7.4	3.64, d, 6.7
2'	5.40, brt, 7.4	5.04, brt, 6.7
4'	1.71, s	1.88, s
5'	2.06, m	1.98, m
6'	2.13, m	2.05, m
7'	5.12, brt, 7.0	5.17, t, 7.0
9'	1.64, s	1.56, s
10'	1.58, s	1.53, s

The NMR data of **4c** correspond well to those published previously (Kumano et al. 2010)

Table S6: ^{1}H-NMR data of compound **5b**

Comp.	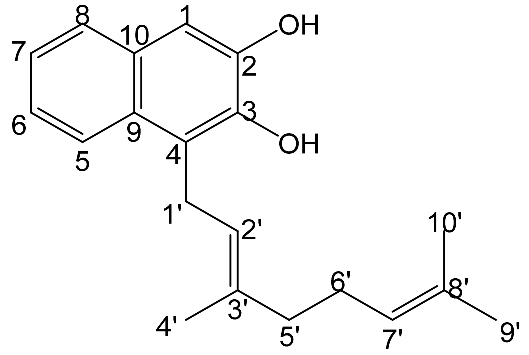 **5b**
Pos.	δ_H, multi, *J* in CD_3OD
1	6.98, s
5	7.52, d, 8.0
6	7.17, t, 6.9
7	7.21, t, 6.9
8	7.71, d, 8.0
1'	3.74, d, 6.7
2'	5.18, brt, 6.7
4'	1.87, s
5'	1.97, m
6'	2.06, m
7'	5.02, brt, 7.2
9'	1.55, s
10'	1.52, s

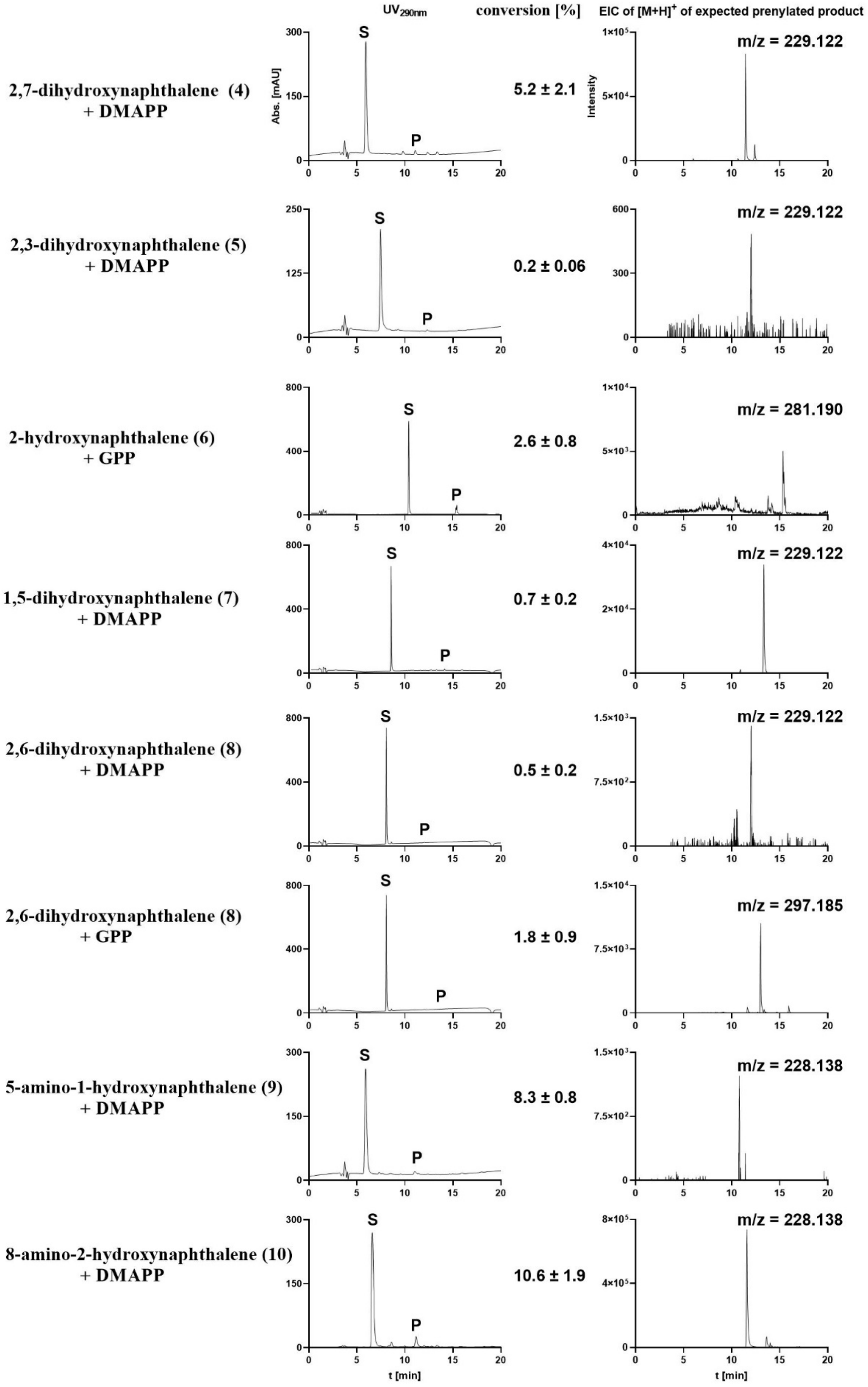

Fig. S1 LC-MS analysis of enzyme assays of ShPT with hydroxynaphthalenes. UV absorptions at 290 nm (left column) and extracted ion chromatography (EICs) for $[M + H]^+$ of the expected monoprenylated product with a tolerance range of ± 0.005 (right column).

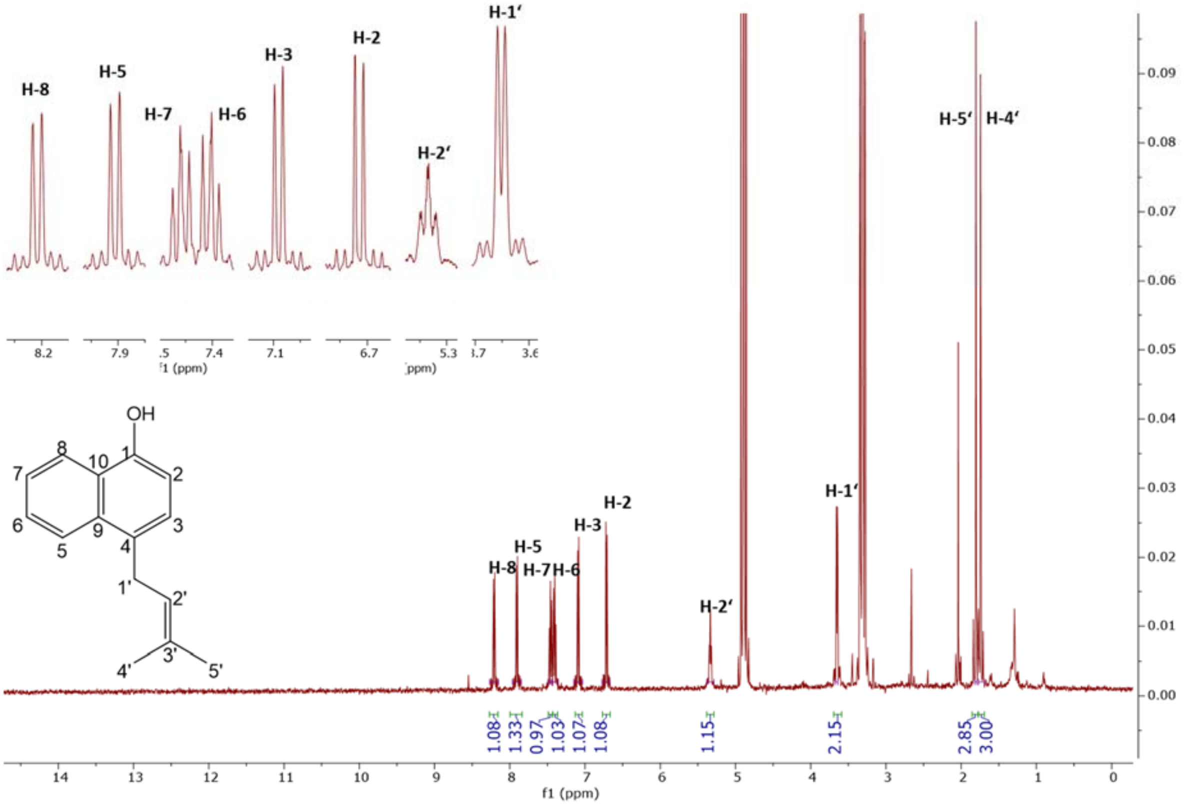

Fig. S2 ^{1}H-NMR spectrum of **1a** in CD_3OD (500 MHz)

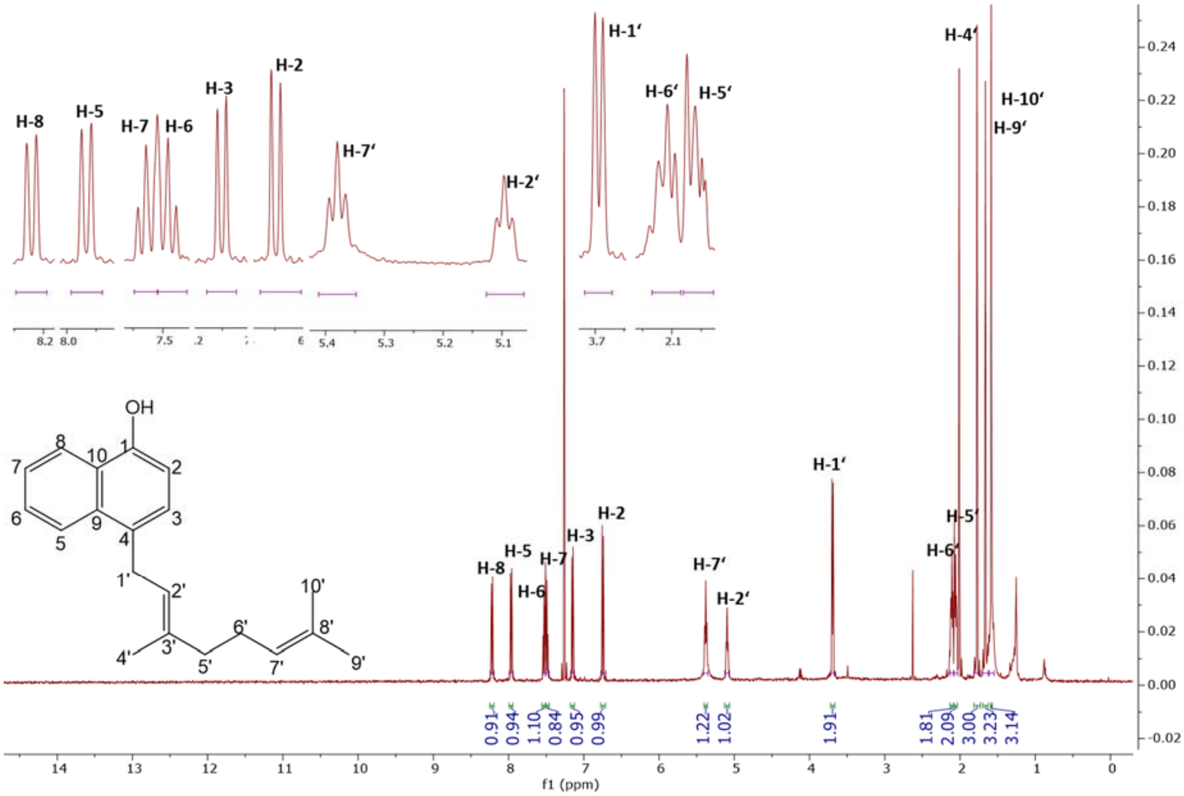

Fig. S3: ^{1}H-NMR spectrum of **1b** in $CDCl_3$ (500 MHz)

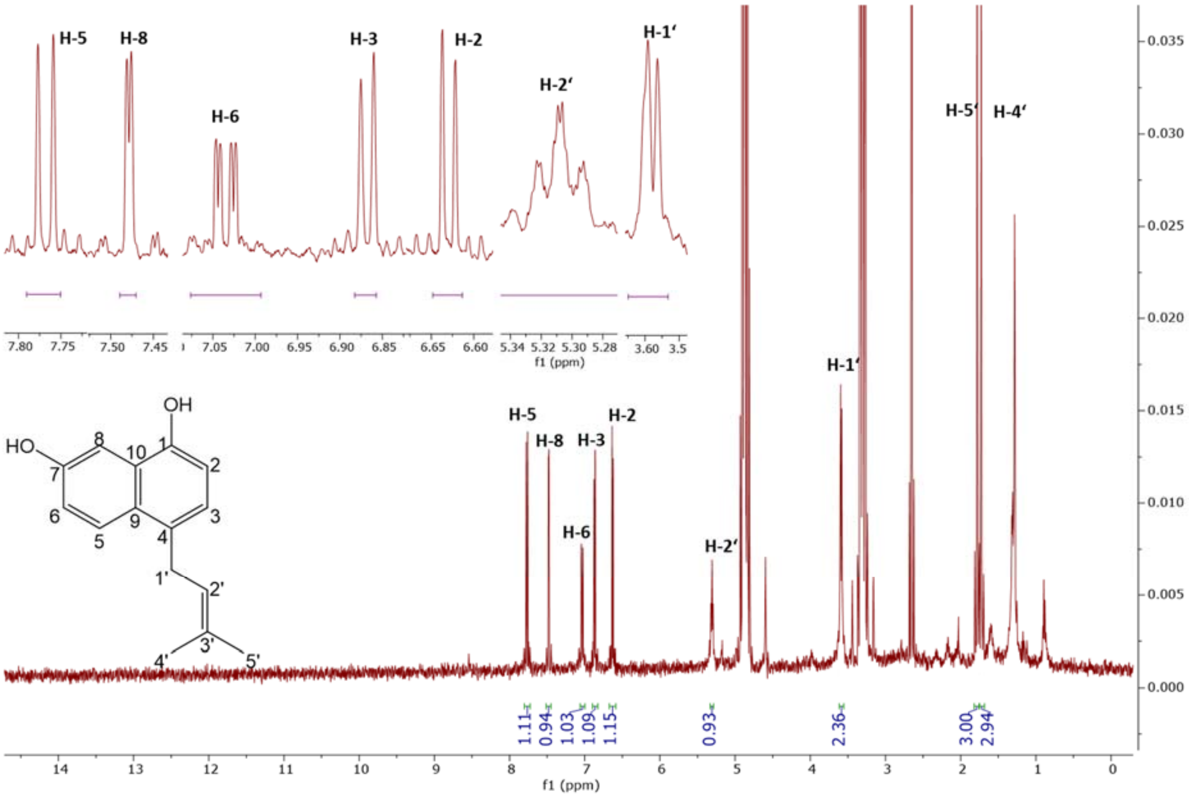

Fig. S4: ^{1}H-NMR spectrum of **2a** in CD_3OD (500 MHz)

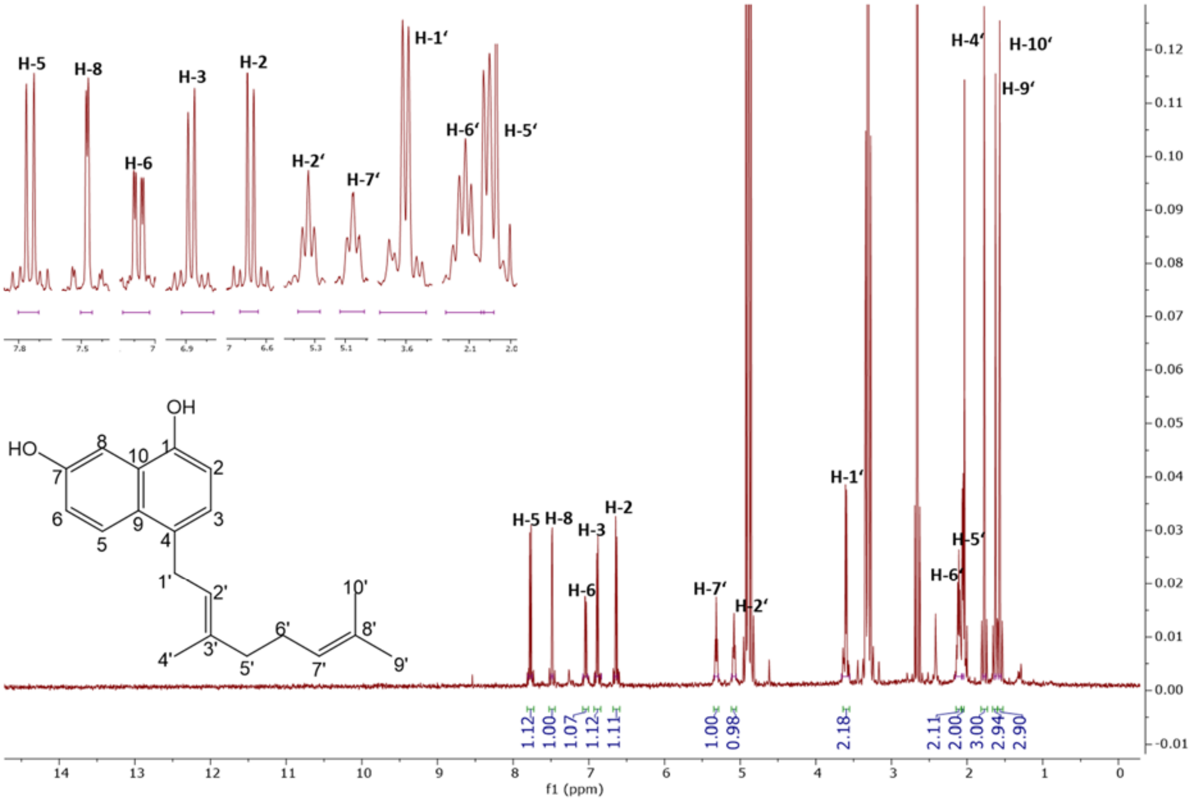

Fig. S5: ^{1}H-NMR spectrum of **2b** in CD_3OD (500 MHz)

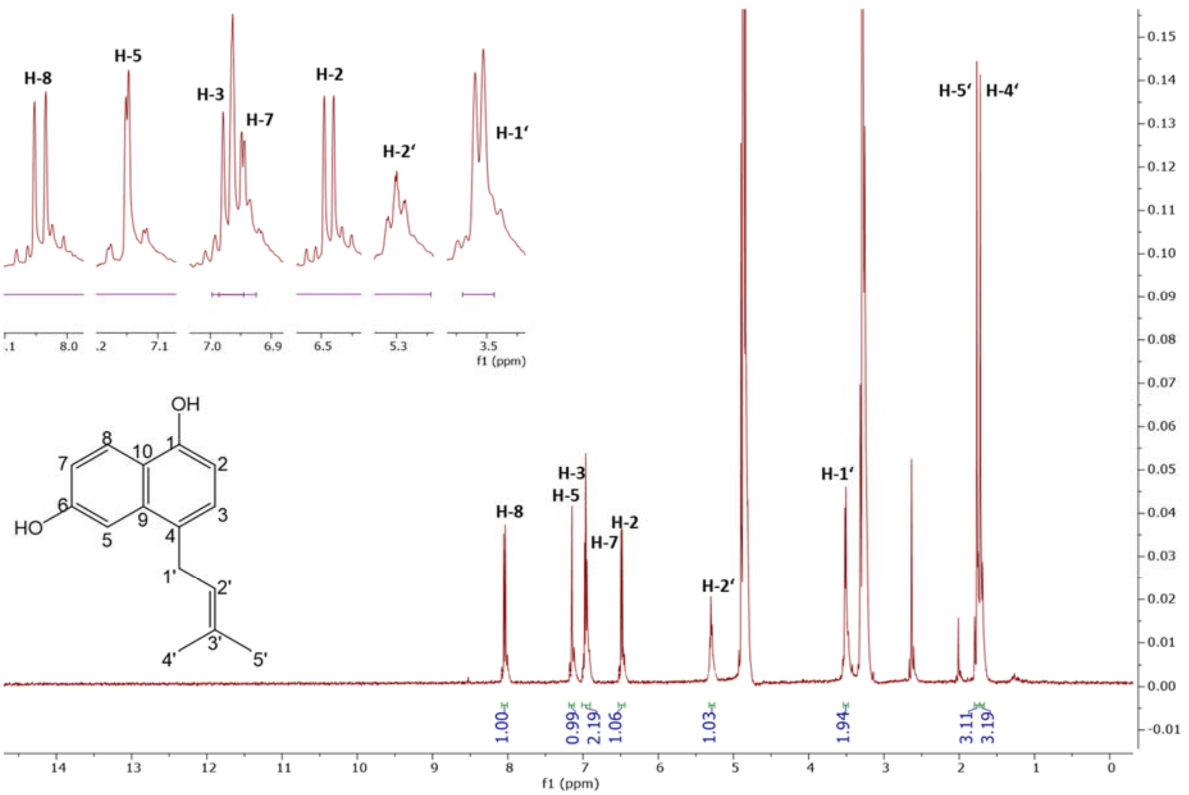

Fig. S6: ^{1}H-NMR spectrum of **3a** in CD_3OD (500 MHz)

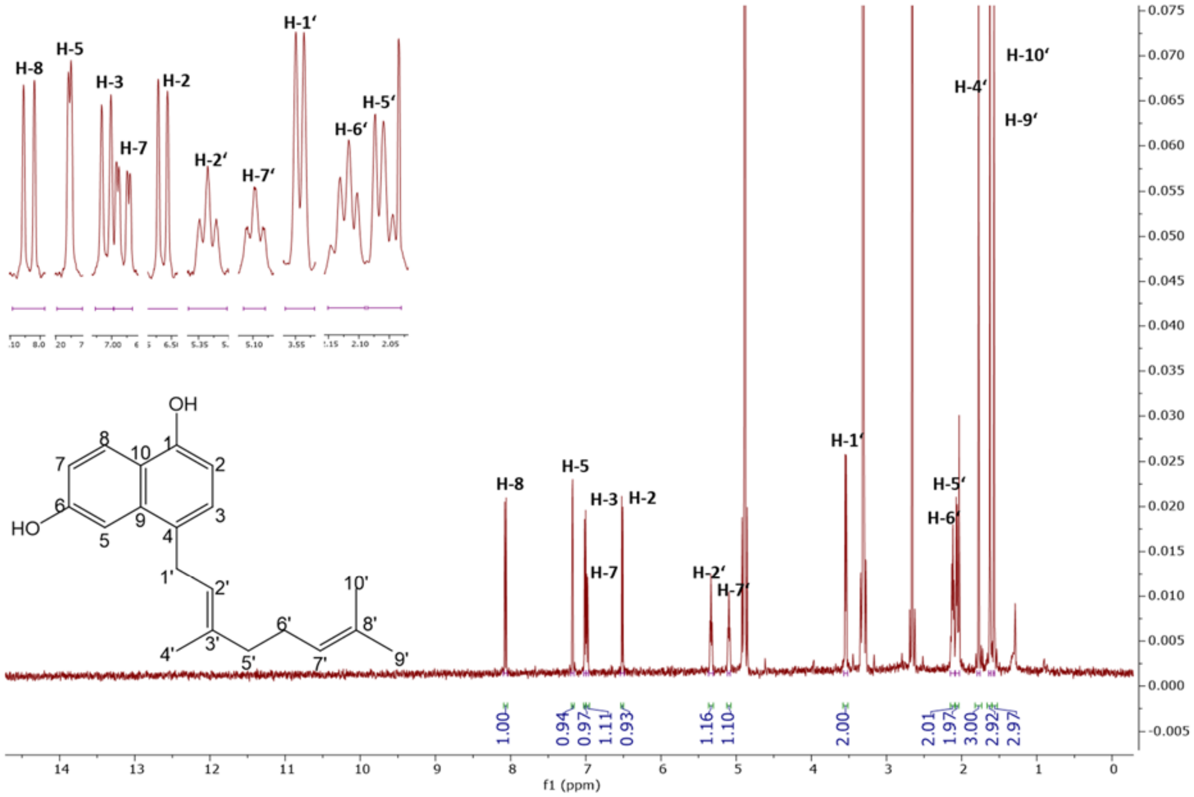

Fig. S7: ^{1}H-NMR spectrum of **3b** in CD_3OD (500 MHz)

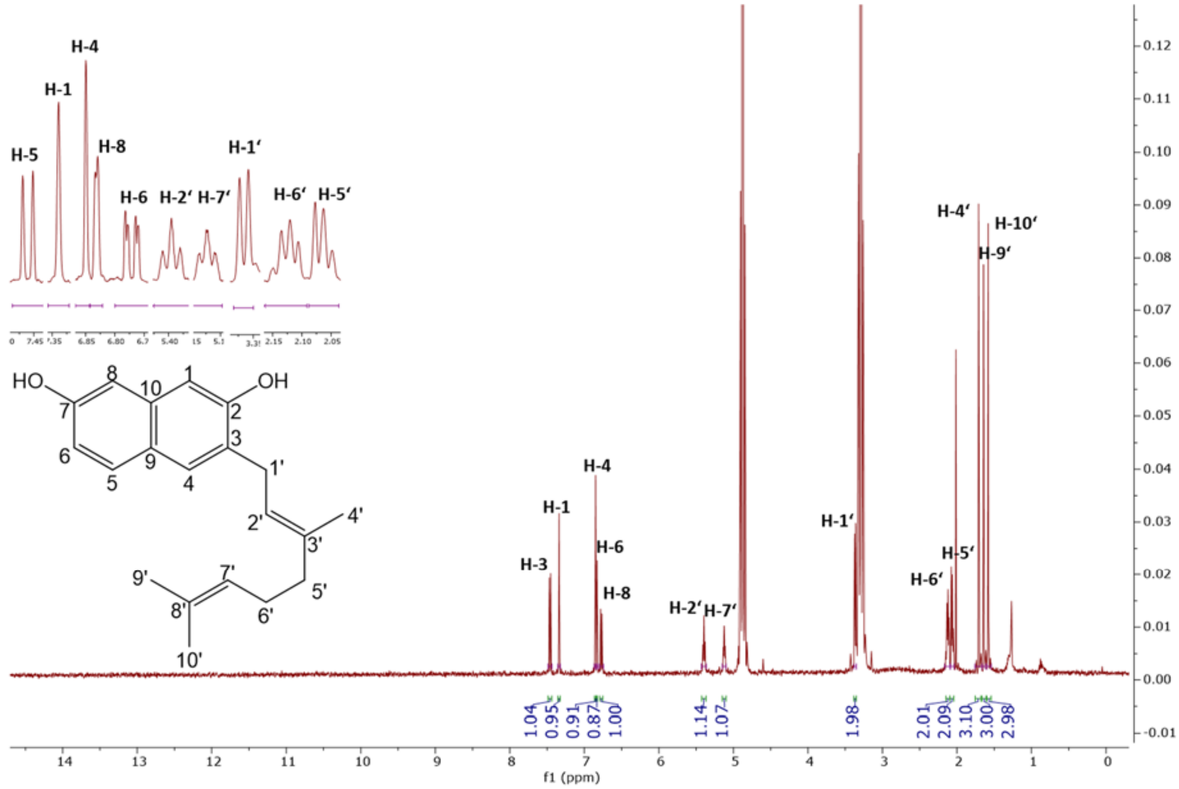

Fig. S8: ^{1}H-NMR spectrum of **4b1** in CD_3OD (500 MHz)

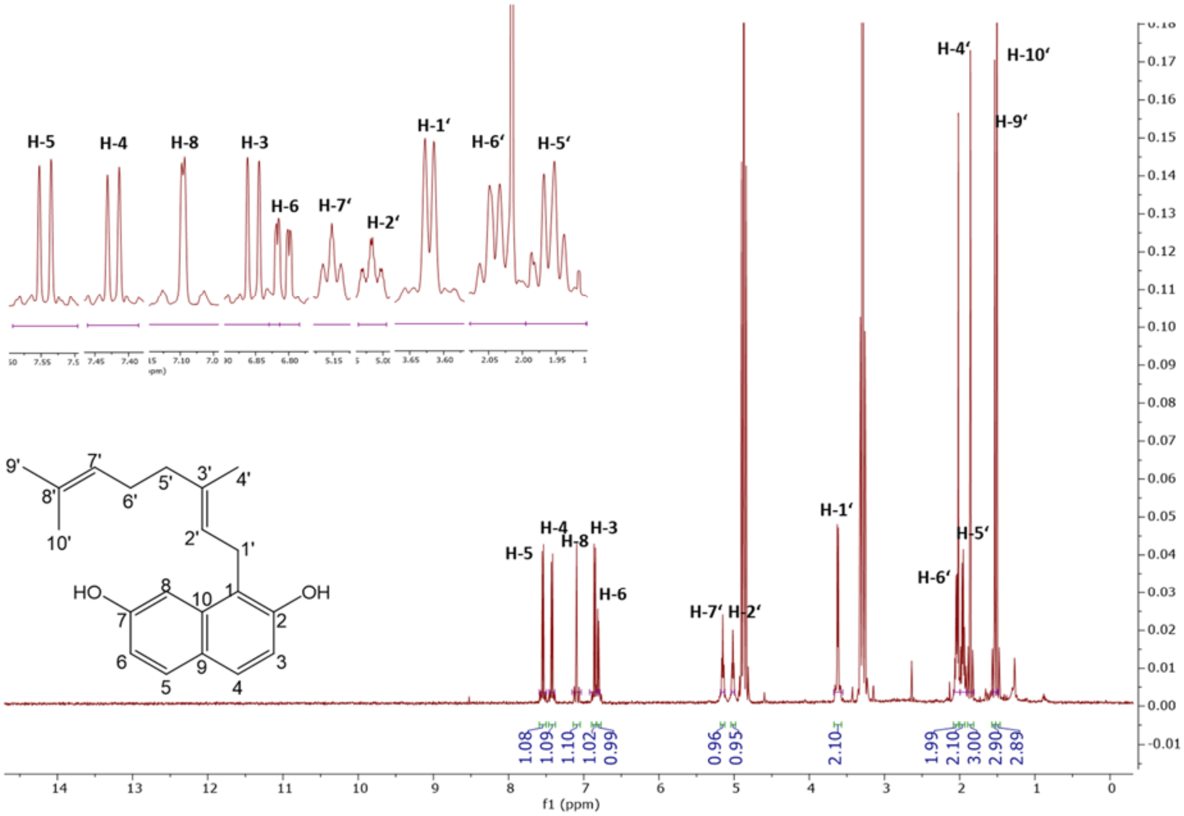

Fig. S9: ^{1}H-NMR spectrum of **4b2** in CD_3OD (500 MHz)

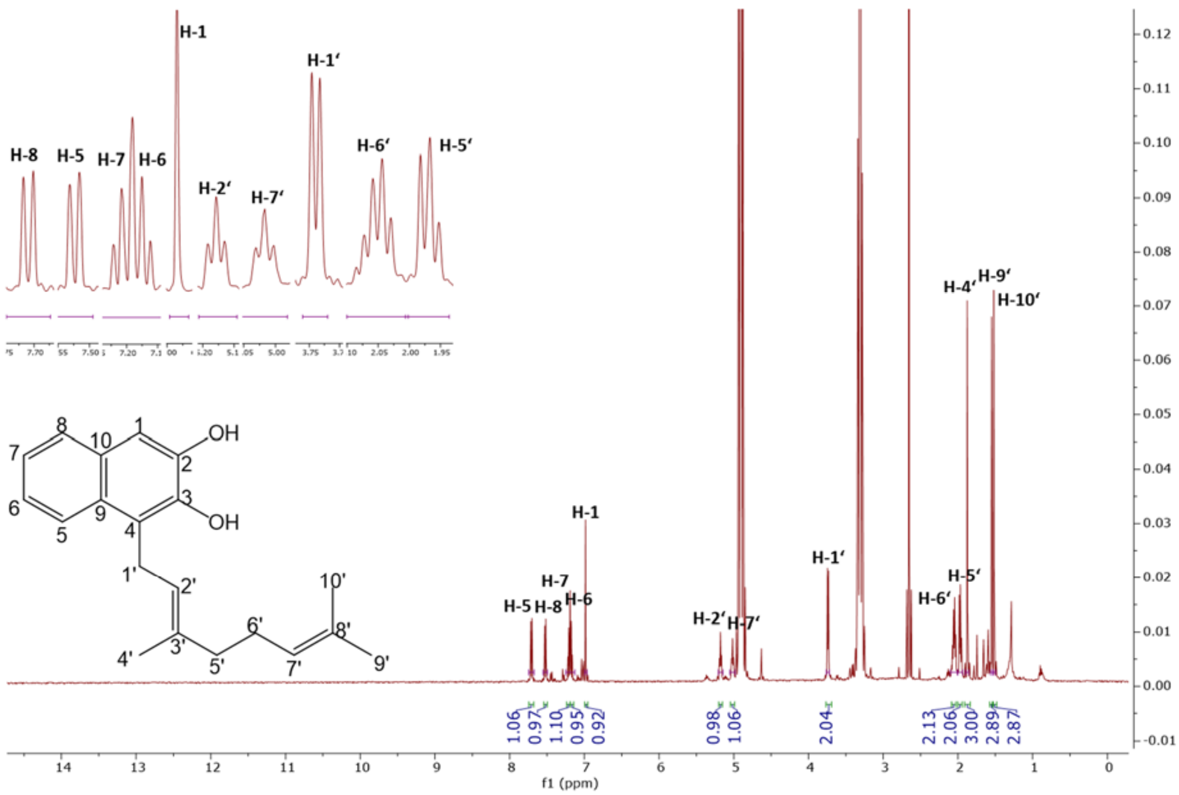

Fig. S10: ^{1}H-NMR spectrum of **5b** in CD_3OD (500 MHz)

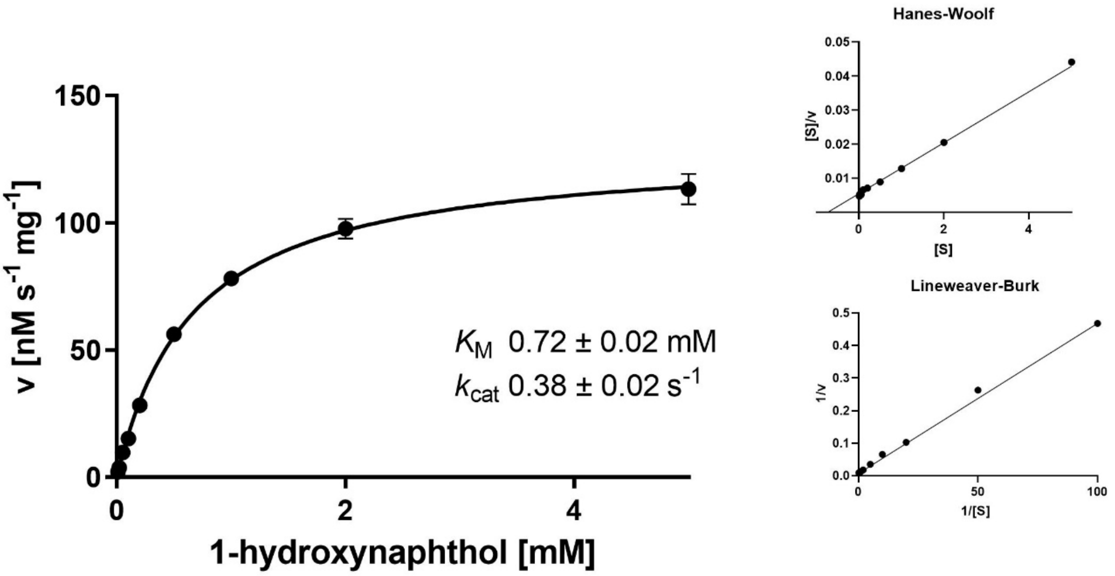

Fig. S11 Kinetic parameters of 1-hydroxynaphthol (**1**) with DMAPP as donor

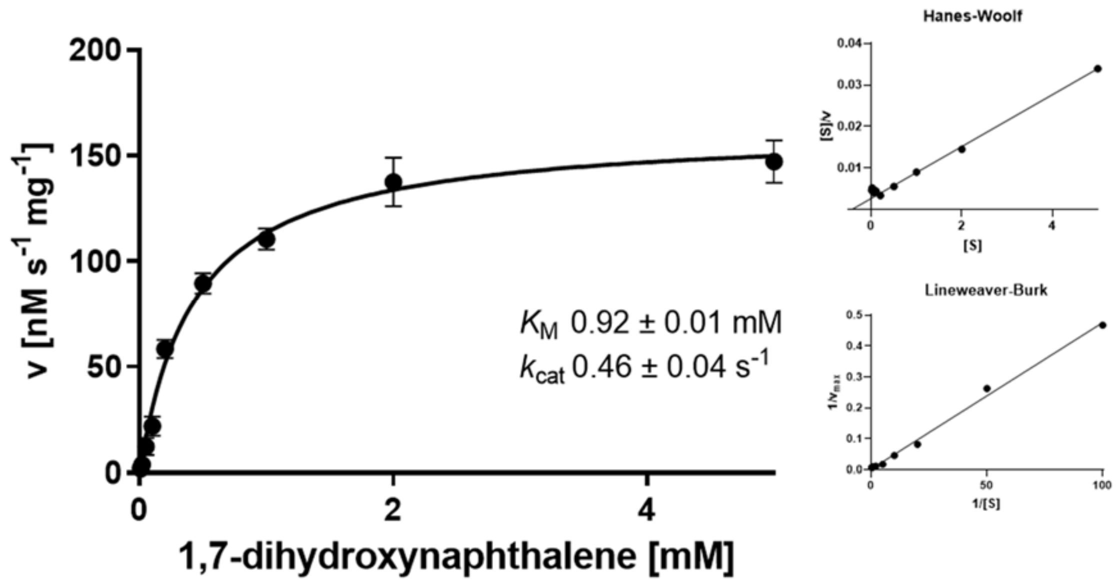

Fig. S12 Kinetic parameters of 1,7-dihydroxynaphthalen (**2**) with DMAPP as donor

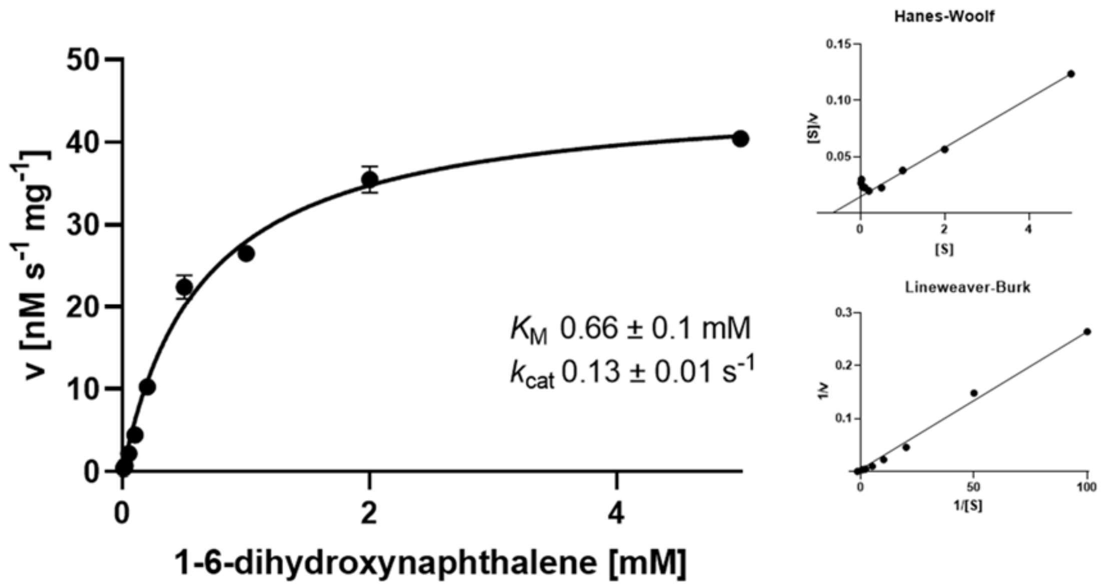

Fig. S13 Kinetic parameters of 1,6-dihydroxynaphthalen (**3**) with DMAPP as donor

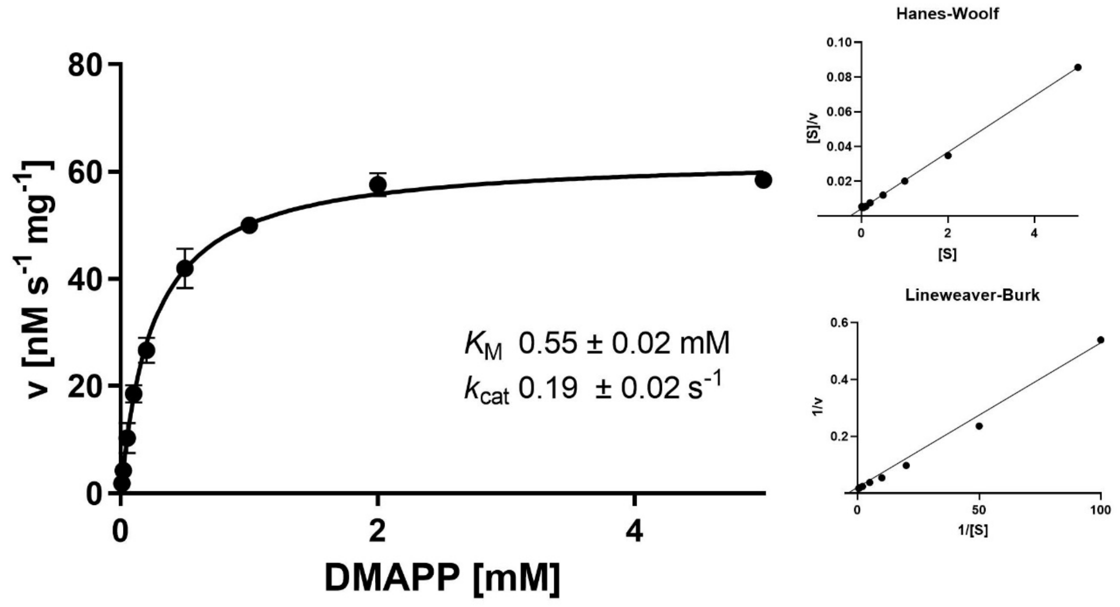

Fig. S14 Kinetic parameters of DMAPP with 1-hydroxynaphthol (**1**) as acceptor

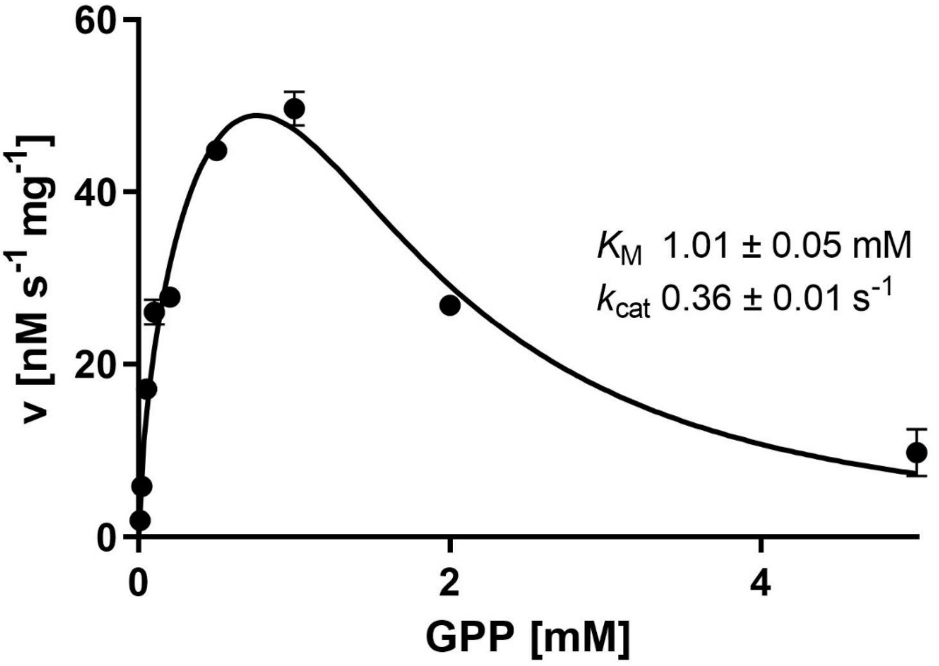

Fig. S15 Kinetic parameters of GPP with 1-hydroxynaphthol (**1**) as acceptor

References

Kumano T, Richard SB, Noel JP, Nishiyama M, Kuzuyama T (2008) Chemoenzymatic syntheses of prenylated aromatic small molecules using Streptomyces prenyltransferases with relaxed substrate specificities. Bioorg. Med. Chem 16:8117–8126. doi: 10.1016/j.bmc.2008.07.052

Kumano T, Tomita T, Nishiyama M, Kuzuyama T (2010) Functional characterization of the promiscuous prenyltransferase responsible for furaquinocin biosynthesis: identification of a physiological polyketide substrate and its prenylated reaction products. J Biol Chem 285:39663–39671. doi: 10.1074/jbc.M110.153957

Törincsi M, Kolonits P, Fekete J, Novak L (2012) Rearrangement of aryl geranyl ethers. Synth.Commun. 42:3187–3199. doi: 10.1080/00397911.2011.579799

Yu X, Xie X, Li S-M (2011) Substrate promiscuity of secondary metabolite enzymes: prenylation of hydroxynaphthalenes by fungal indole prenyltransferases. App. Microbol. Biotechnol. 92:737–748. doi: 10.1007/s00253-011-3351-y
